# Muscle contractility in spinobulbar muscular atrophy

**DOI:** 10.1038/s41598-019-41240-y

**Published:** 2019-03-18

**Authors:** Julia R. Dahlqvist, Sofie T. Oestergaard, Nanna S. Poulsen, Kirsten Lykke Knak, Carsten Thomsen, John Vissing

**Affiliations:** 1 Copenhagen Neuromuscular Center, section 3342 Department of Neurology, Rigshospitalet, University of Copenhagen Blegdamsvej 9, 2100 Copenhagen, Denmark; 2 Department of Radiology, Rigshospitalet, University of Copenhagen Blegdamsvej 9, 2100 Copenhagen, Denmark

## Abstract

Spinobulbar muscular atrophy (SBMA) is caused by a trinucleotide repeat expansion in the androgen receptor gene on the X chromosome. There is a toxic effect of the mutant receptor on muscle and neurons resulting in muscle weakness and atrophy. The weakness can be explained by wasting due to loss of muscle cells, but it is unknown whether weakness also relates to poor muscle contractility of the remaining musculature. In this study, we investigated the muscle contractility in SBMA. We used stationary dynamometry and quantitative MRI to assess muscle strength and absolute and fat-free, cross-sectional areas. Specific muscle force (strength per cross-sectional area) and contractility (strength per fat-free cross-sectional area) were compared with healthy controls and their relation to walking distance and disease severity was investigated. Specific force was reduced by 14–49% in SBMA patients compared to healthy controls. Contractility was reduced by 22–39% in elbow flexion, knee extension, ankle dorsi- and plantarflexion in SBMA patients. The contractility decreased with increasing muscle fat content in muscles with affected contractility in SBMA. The decreased muscle contractility in SBMA may relate to motor neuron degeneration and changed fibre type distribution and muscle architecture.

## Introduction

Spinobulbar muscular atrophy (SBMA) is an X-linked inherited neuromuscular disease caused by a trinucleotide (CAG) expansion in the androgen receptor gene^1^. It is characterized by adult-onset muscle weakness and atrophy of bulbar and limb muscles. Historically, it was believed that the motor dysfunction was caused solely by affection of lower motor neurons and denervation of the muscle due to a toxic gain of function of the mutated androgen receptor upon binding with testosterone. However, research in the last decade suggests that the mutant androgen receptor also causes a direct toxic effect on muscle^1^.

In the development of new treatments for patients with SBMA it is important to understand why their muscles are weak. Muscle strength depends on the muscle mass, and a validated surrogate of muscle mass is the cross-sectional area (CSA) perpendicular to the direction of muscle fibers^2^. The relation between muscle strength and CSA is linear in healthy individuals and independent of sex^2^. In SBMA, there is neurogenic muscle atrophy and fat replacement, which together result in reduced muscle CSA^3^. Both muscle atrophy and fat replacement contribute to the weakness seen in SBMA. However, the muscle contractile quality might also be affected in SBMA due to the myotoxicity of the mutated androgen receptor and its tendency to aggregate in myocytes, which affects cellular signalling transduction and mitochondrial function^4^.

In this study, we investigated the characteristics of the muscle contractile quality in patients with SBMA. We used stationary dynamometry to assess muscle strength and quantitative Dixon MRI to calculate absolute CSAs and fat-free contractile CSAs (CCSA) and compared the quality in SBMA to healthy controls. We also investigated the relation between the contractile quality and CAG-repeat length, age, disease duration, disease severity, and walking distance in patients with SBMA.

## Methods

### Ethical approval

The research protocol was approved by the Danish National Committee on Health Research Ethics (approval number: H-15006316) and was registered at Clinicaltrials.gov (Identifier: NCT02501395). Informed consent was obtained from all participants, and the study was conducted in accordance with the declaration of Helsinki.

### Study design and participants

In this observational and cross-sectional study, we investigated patients and healthy controls at the Copenhagen Neuromuscular Center, Rigshospitalet, Denmark from September 2015 to May 2018. We investigated 39 patients with SBMA and 40 healthy controls. All SBMA patients had a genetically confirmed diagnosis. Exclusion criteria were contraindications to MRI and competing disorders that could interfere with the results. Patients were recruited from the Copenhagen Neuromuscular Center and controls from the local community. Some of the data from healthy volunteers has been published before and the patients were enrolled in a previous MR study^5^.

### MRI

We scanned the legs and the right arm of all participants using a 3.0 T Siemens scanner (MAGNETOM Verio Tim System; Siemens AG, Erlangen, Germany). The MRI protocol included localizers, T1-weighted images and 2-point Dixon sequences as described before^6^.

The images were analysed by two examiners using Numaris/4 B17 and Osirix MD software. Inter-observer agreement was 91% ± 1.8%. We mapped muscles or muscle groups on four defined Dixon slices with the greatest cross-sectional areas of most muscles: one corresponding to 50% of the length of femur, one to 33% of the length of tibia and the last two at the widest parts of the upper arm and forearm^6^. The muscle mapping generated information about CSA and fat and water signals, and fat fractions were calculated by expressing the fat signal as a percentage of the total water and fat signal. We also calculated a fat-free CSA, or a contractile-CSA (CCSA), by multiplying the CSA by one minus the fat fraction.

### Muscle strength

We tested maximal isokinetic peak torque of elbow flexion and extension, knee extension and flexion, ankle plantarflexion and dorsiflexion on the right side of all participants using a stationary dynamometer (Biodex System PRO 4 dynamometer®, Biodex Medical Systems, NY, USA). The tests consisted of 8 repeats of muscle contraction with 15 seconds of rest between each contraction.

We calculated specific muscle force (muscle strength/CSA) and muscle contractility (muscle strength/CCSA).

Muscle fatigability in knee extensors was tested in the stationary dynamometer using an isometric knee extension test. The test consisted of 60 repeats of 3 seconds of muscle contraction with 2 seconds of rest between each contraction. The participants were instructed to exert maximum force during each contraction.

### Clinical evaluation of patients

Body mass index was calculated. Muscle function in patients with SBMA were assessed using the validated Spinal and Bulbar Muscular Atrophy Functional Rating Scale (SBMAFRS) and the 6-minute walk test (6MWT)^7^. All patients completed the validated, self-administered Fatigue Severity Scale that investigates the severity of fatigue and its impact on daily function^8^. The total score ranges from 1 (no fatigue) to 7 and the cut-off score for fatigue is >4^9^.

### Statistical analyses

Statistical analysis was performed using SPSS v25 and values are mean ± standard deviation (SD) unless otherwise stated. A p-value of <0.05 was considered statistically significant.

ANOVA was used for comparing fat content, CSA, CCSA, muscle strength, muscle strength/CSA, and muscle strength/CCSA among patients with SBMA and controls. P-values were Bonferroni-corrected. Pearson correlation and linear regression were used for measuring correlation between parameters. When data were not normally distributed or had unequal variance, logarithm transformation was performed.

## Results

### Participants

Please see Table 1 for participant characteristics. There was no difference in age and body mass index between patients and healthy controls (*P* > 0.51). Four patients with SBMA were wheelchair bound and 11 patients used walking aid (cane, crutches or walker).

**Table 1:** Participant characteristics.

	SBMA		Healthy controls	
	mean	(range)	mean	(range)
Sex (m/f)	39/0		27/13	
Age (years)	58.2	(26–83)	53.8	(27–81)
BMI (kg/m^2^)	24.9	(18.3–31.4)	25.7	(19.3–32.3)
CAG-repeat length (number)	43.3	(38–52)	*ND*	
SBMAFRS (0–56, worse to normal)	42.9	(25–56)	*ND*	
6-minute walk test (meter)	409	(54–800)	*ND*	

SBMA, Spinobulbar muscular atrophy; BMI, body mass index; SBMAFRS, Spinal and Bulbar Muscular Atrophy Functional Rating Scale; ND, not done.

**Figure 1:**
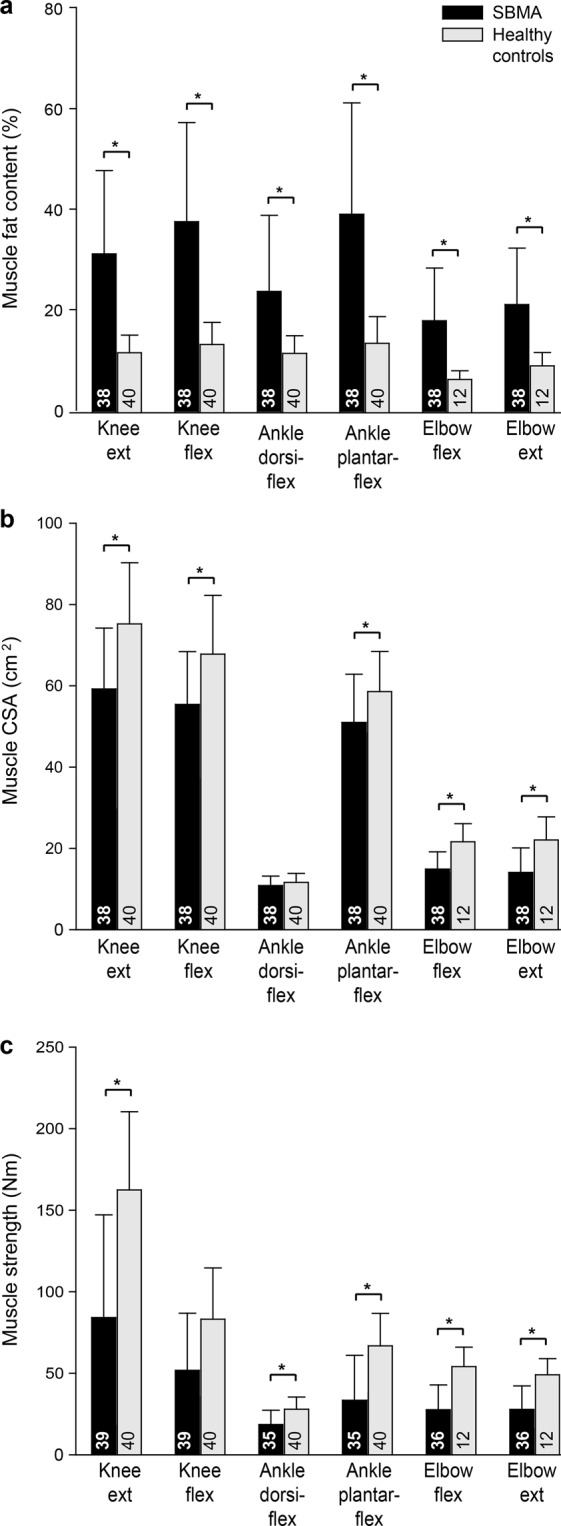
Muscle fat content (**a**), cross-sectional areas (**b**), and muscle strength (**c**) in patients with spinobulbar muscular atrophy and healthy controls. CSA, cross-sectional area; ext., extensors; flex., flexors. Numbers in bars represent the number of participants. Asterisks indicate significant difference.

Not all patients were included in all analyses. Patients that were too weak to perform the muscle strength tests at one or more levels were not included in the respective analyses. Also, one patient with SBMA did not have back and leg MRI sequences performed due to claustrophobia, but had arm MRI sequences performed. Please see Figs 1 and 3 for number of participants in each analysis.

### Muscle fat content, cross-sectional area and strength

Compared to healthy controls, patients with SBMA had higher fat content (*P* < 0.001; Fig. 1a) and decreased muscle strength in all tested muscles (*P* < 0.0002; Fig. 1c). Muscle CSAs were smaller for thigh muscles, ankle plantar flexors and upper arm muscles (*P* < 0.01; Fig. 1b), but not for ankle dorsiflexors (*P* = 0.38).

**Figure 2:**
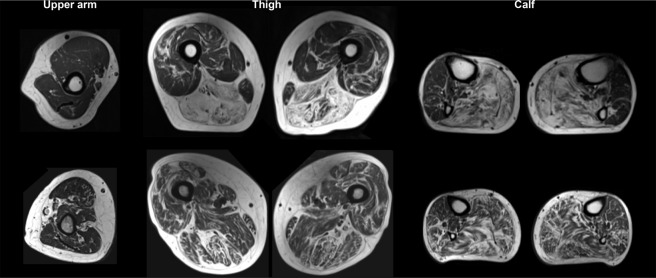
MR images of upper arm, thigh and calf muscles in one patient with spinobulbar muscular atrophy T1-weighted MR images of the upper arm, thigh and calf in two patients with spinobulbar muscular atrophy.

The most affected muscles in patients with SBMA were the posterior muscles of the thighs and calves, while the sartorius, gracilis and tibialis anterior muscles were the most spared (Fig. 2).

### Specific muscle force

Patients with SBMA had lower specific muscle force in all examined muscle groups (*P* < 0.004) except the elbow extensors (*P* = 0.20) compared to healthy controls.

The relation between muscle strength and the CSA was linear for knee extensors (R 0.81; *P* < 0.0001), knee flexors (R 0.51; *P* = 0.001), elbow extensors (R 0.84; *P* < 0.0001), and elbow flexors (R 0.80; *P* < 0.0001), but not for ankle dorsi- and plantar flexors (R < 0.25; *P* > 0.16) in patients with SBMA. In healthy controls, it was linear in all muscles (R 0.44–0.82; *P* < 0.05).

There was a linear relation between specific muscle force and muscle fat content in all muscles (R 0.66–0.92; *P* < 0.0001) except elbow extensors in the patients with SBMA, and in elbow extensors, knee extensors and ankle dorsiflexors in the healthy controls (R 0.33–0.61; *P* < 0.05). These results indicate that muscles with high fat content can generate less force than muscles with lower fat content, which is expected.

### Muscle contractility

**Figure 3:**
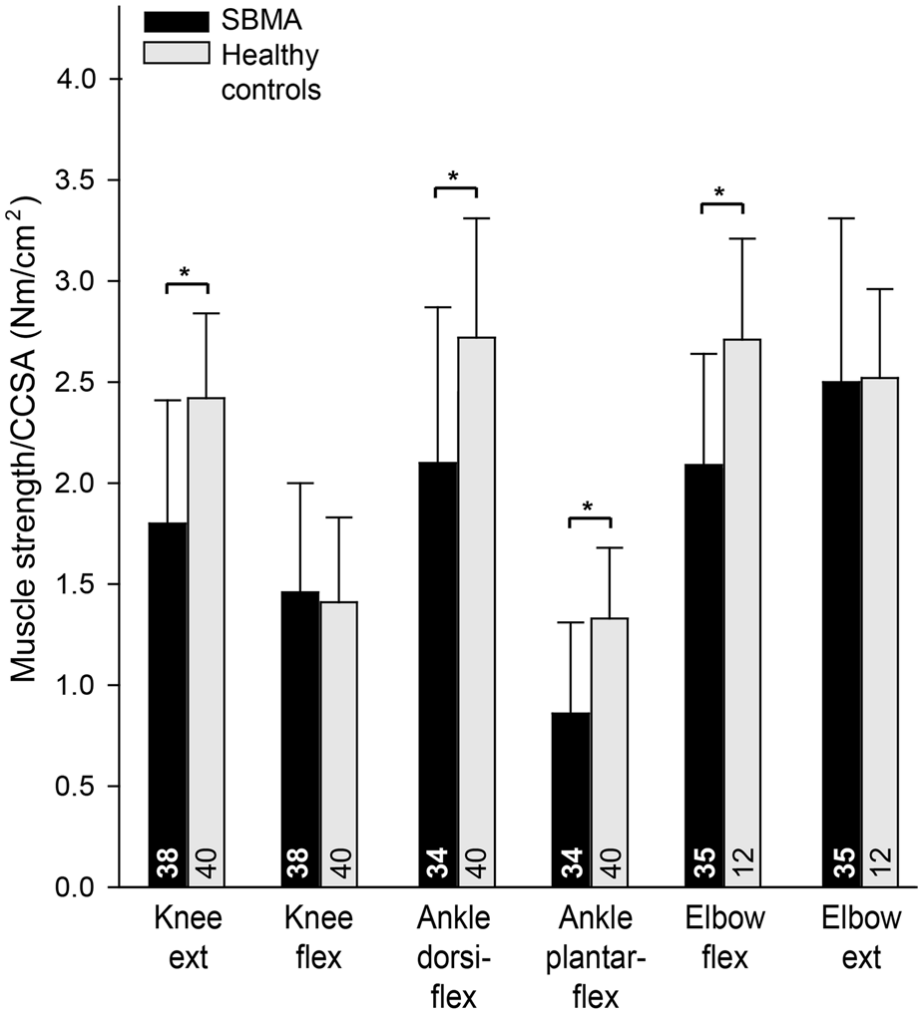
Contractility in patients with spinobulbar muscular atrophy and controls. CSA, cross-sectional area; CCSA, contractile cross-sectional area; ext., extensors; flex., flexors. Numbers in bars represent the number of participants. Asterisks indicate significant difference.

The muscle contractility was lower in knee extensors, ankle plantar- and dorsiflexors, and elbow flexors in patients with SBMA compared to healthy controls (*P* < 0.004; Fig. 3).

**Figure 4:**
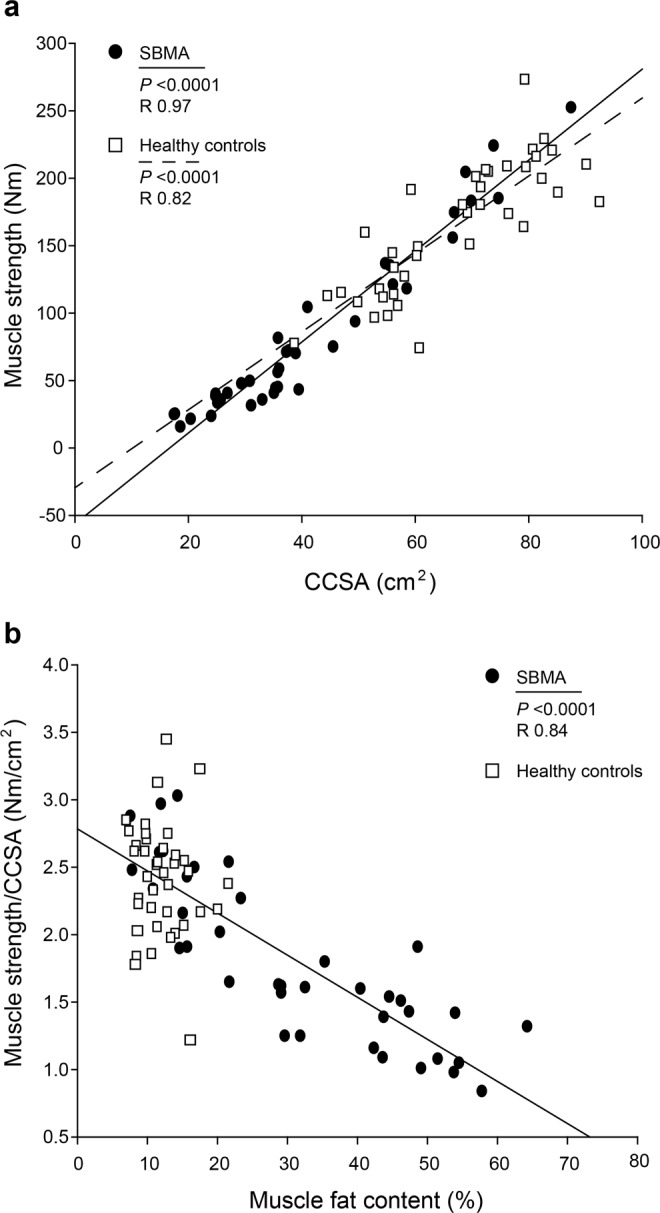
Correlations between muscle strength and contractile cross-sectional areas in knee extensors (**a**) and between muscle strength per contractile cross-sectional area and muscle fat content (**b**) in knee extensors of patients with spinobulbar muscular atrophy and healthy controls. Regression lines indicate significant correlations (dashed line, healthy controls; solid line, patients with SBMA). Significant *P*- and R-values are specified in the graphs. CCSA, contractile cross-sectional area.

The relation between muscle strength and the CCSA was linear in all muscles in both patients with SBMA and healthy controls (R 0.43–0.97; *P* < 0.05; Fig. 4a).

To investigate whether the muscle contractility was affected by muscle fat content, we tested the linear relationship between the two (Fig. 4b). The relation was linear in elbow flexion (R 0.46; *P* = 0.005), knee extension (R 0.84; *P* < 0.0001), and ankle dorsi- (R 0.56; *P* = 0.001) and plantarflexion (R 0.85; *P* < 0.0001) in patients with SBMA, but not in knee flexion (*P* = 0.78) and elbow extension (*P* = 0.06).

### Muscle fatigability

**Figure 5:**
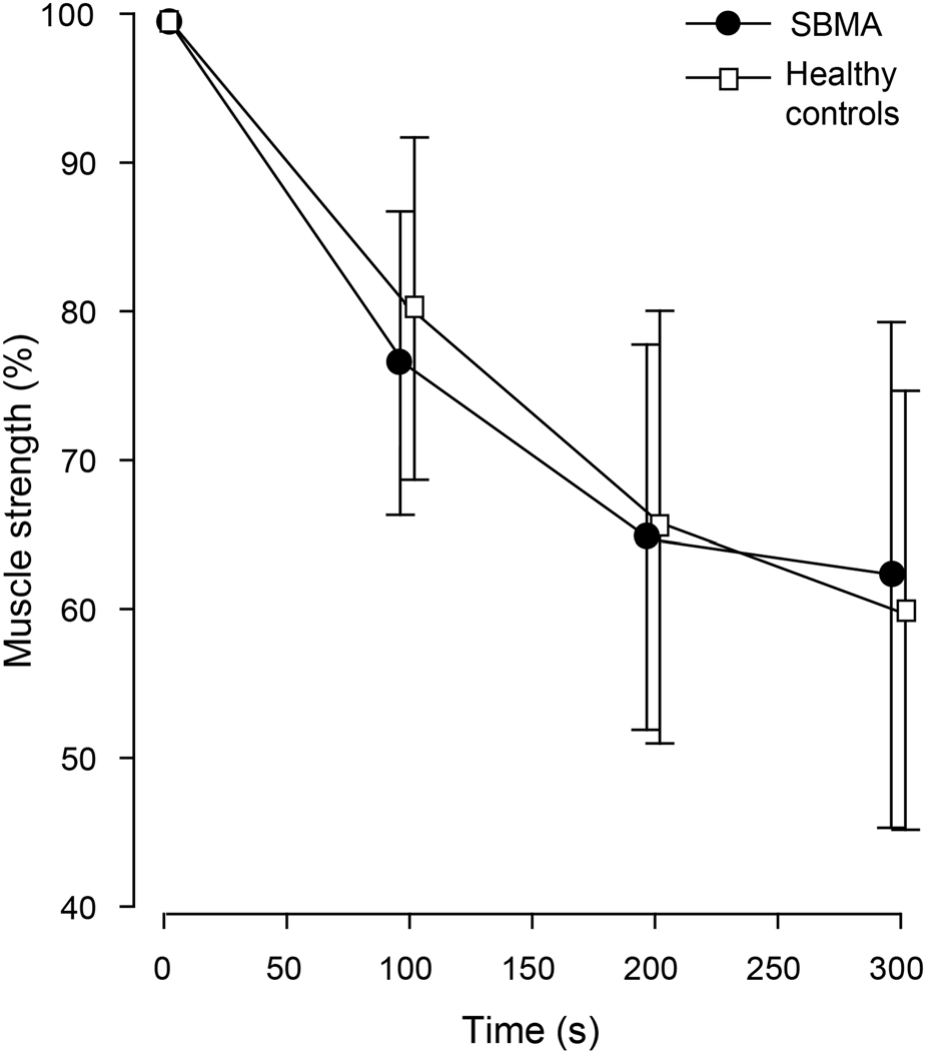
Reduction in strength from first repeat in the intermittent knee extension test in patients with spinobulbar muscular atrophy and healthy controls.

We examined muscle fatigability during repeated knee extension contractions in 7 patients with SBMA and 7 healthy controls (Fig. 5). Patients with SBMA had a reduction in knee extension strength of 38 ± 17% (from 99 ± 50 Nm to 59 ± 32 Nm), and healthy controls 40 ± 15% (from 190 ± 89 to 109 ± 45). There was no difference in fatigability between groups (*P* = 0.88).

In the Fatigue Severity Scale, patients with SBMA had a mean score of 5.1 ± 1.5 compared to 3.0 ± 1.1 in healthy controls^9^. Fatigue (score ≥ 4) was seen in 28 patients (73%) with SBMA. For comparison, it has been reported in 18% of healthy subjects^9^.

### Correlations among muscular, clinical and personal features

In healthy controls, the specific muscle force decreased with age in knee extensors and -flexors and ankle dorsiflexors (*R* 0.34–0.57*; P* < 0.05), and the contractility in ankle dorsiflexors (*R* 0.53*; P* < 0.001). In patients with SBMA, the specific muscle force decreased with age in all muscles except elbow extensors (*R* 0.46–0.67; *P* < 0.001), and the contractility in knee extensors, ankle plantar- and dorsiflexors, and elbow flexors decreased (*R* 0.44–0.64*; P* < 0.05). Body mass index did not correlate with specific force or contractility in patients and controls (*P* > 0.75).

Disease duration correlated with specific force and contractility in the patients. The specific force decreased with disease duration in all muscles except elbow extensors (*R* 0.33–0.55*; P* < 0.05). For muscle contractility, knee extensors, ankle plantar flexors, and elbow flexors decreased (*R* 0.39–0.51*; P* < 0.05).

Stronger correlations were seen among specific force, contractility and muscle function. As a mean specific force of the examined leg muscles decreased, the SBMAFRS score (*R* 0.78*; P* < 0.0001) and 6MWT distance (*R* 0.85*; P* < 0.0001) also decreased. The same was seen for the mean contractility of the leg muscles; SBMAFRS score (*R* 0.69*; P* < 0.0001) and 6MWT distance (*R* 0.77*; P* < 0.0001) decreased with decreasing contractility.

## Discussion

In this study, we investigated the specific muscle force (the muscle strength per CSA) and the muscle contractility (the muscle strength per fat-free contractile CSA) in patients with SBMA and compared it to healthy controls. We found that the specific force and muscle contractility was decreased in patients with SBMA compared to healthy controls. The muscle contractility decreased with increasing muscle fat content in elbow flexors, knee extensors and ankle dorsi- and plantar flexors in the patients with SBMA.

There is an obvious loss of muscle strength in most neuromuscular disorders when the muscles become atrophic and are replaced by fat and it is therefore not surprising that we found decreased specific muscle force in patients with SBMA. However, we also found that the contractility of the remaining musculature was decreased. Muscle contractility is influenced by muscle architecture, composition and metabolism^10^, and it has previously been shown that myopathies with abnormal architecture, composition and/or metabolism have decreased muscle contractility^11–13^. Patients with SBMA have a muscle histopathology with myopathic changes, including hypertrophic fibres, myofibrillar disorganization and depletion of mitochondria, which can explain some of the reduced contractility^14^.

The muscle contractility in our patients with SBMA decreased with increasing muscle fat content in all muscle groups that had affected muscle contractility. This finding suggests that the muscle fat has a negative effect on the surrounding muscle tissue, which is in agreement with findings in healthy, elderly subjects^10^. The age-related muscle mass loss and conversion into fat tissue in healthy is partly caused by motor neuron loss, which is also seen in SBMA^15^. The muscle fat is not just inactive tissue that fills the space where muscles are loss^10^. Excessive amounts of muscle fat seem to negatively affect the muscle tissue and is associated with impaired physical performance, decreased walking distance, and decreased mobility^16^. Further, it has been shown that increases in muscle fat content lead to the transition of muscle fibres from fast-twitch, glycolytic type II fibres to slow-twitch, oxidative type I fibers^10^. Since type I fibres have decreased muscle force and contractility compared to type II fibres this transition probably results in muscles with decreased force and contractility^17^. The same transition of fibre types has been seen in muscles of patients with SBMA^18^. Thus, the increased muscle fat content in SBMA probably affects muscle contractility both directly and indirectly and may explain some of the decreased contractility.

The fat replacement of muscle in SBMA showed a mottled pattern with irregular arrangement of fat and muscle tissue within the muscles. This is different from the pattern seen in muscular dystrophies where the fat replacement is more localized in certain muscles or parts of a muscle. We believe that the spotty pattern of fat distribution in SBMA is caused by the loss of motor neurons innervating random groups of muscle fibres in each muscle. The motor neuron degeneration in SBMA probably explains some of the decreased contractility since affected neuronal activation also affects contractility^10^. Motor neuron degeneration is also associated with muscle fatigue^19^. Activity-induced fatigue, which is caused by repetitive activity, has been demonstrated in patients with SBMA and Spinal muscular atrophy^19–22^. The surviving nerves in SBMA show collateral sprouting resulting in enlarged motor units, which are mechanically less efficient and show increased fatigability compared to motor units in healthy subjects^23^. However, in our repeated knee extension test, we found that muscle fatigability was similar in patients with SBMA and healthy controls. As discussed below, this may be explained by the short duration and high intensity of the test.

A more general fatigue is also a common complaint in patients with SBMA^19^. In the fatigue severity score, 73% of our patients with SBMA reported fatigue. The item with the highest score for SBMA patients was ‘exercise brings on my fatigue’. We have previously shown that aerobic exercise at moderate intensity was not well tolerated in patients with SBMA and may even promote fatigue^24^. Twelve weeks of cycle training did not improve maximal oxygen uptake or ADL functions^24^. With regard to the fatigue, exercise of shorter duration could be beneficial for patients with SBMA and, recently, it has been shown that 8 weeks of high-intensity, short-duration interval training is well tolerated and improves fitness in patients with SBMA^25^. Our repeated knee extension test was of high-intensity and short duration and was also well tolerated by patients with SBMA and without muscle fatigue greater than healthy controls. Thus, new studies of fatigue in SBMA are warranted to investigate if fatigue in SBMA relates to specific muscle groups or contraction patterns and intensities.

In conclusion, we demonstrate widespread decreased muscle contractility in patients with SBMA compared to healthy controls. The decreased contractility in SBMA may relate to several underlying factors including motor neuron degeneration and changed fibre type distribution, muscle architecture and metabolism.

## Data Availability

The datasets generated during and/or analysed during the current study are available from the corresponding author on reasonable request.
